# Prostatic Relaxation Induced by Loperamide Is Reduced in Spontaneously Hypertensive Rats

**DOI:** 10.1100/2012/941685

**Published:** 2012-05-03

**Authors:** Liang-Ming Lee, Chih-Cheng Lu, Hsien-Hui Chung, Juei-Tang Cheng

**Affiliations:** ^1^Department of Urology, College of Medicine, Wan-Fang Hospital, Taipei Medical University, Wang-Fang, Taipei 110, Taiwan; ^2^Department of Surgery, Chi Mei Medical Center Liouying, LiouYing, Tainan 736, Taiwan; ^3^Institute of Basic Medical Sciences, College of Medicine, National Cheng Kung University, Tainan 701, Taiwan; ^4^Department of Medical Research, Chi Mei Medical Center, Yong Kang, Tainan 710, Taiwan

## Abstract

This paper shows a new finding about the decrease of relaxative response to loperamide in prostate of spontaneously hypertensive rats (SHR) as compare to normal rats (WKY). Authors demonstrated the reduction of ATP-sensitive potassium channels is resposible for this change using immunoblotting analysis and the decrease of action induced by diazoxide. This view is not mentioned before and is the first one reporting this result.

## 1. Introduction

Benign prostatic hyperplasia (BPH) occurs frequently in older men and is correlated with lower urinary tract symptoms causing obstruction of the proximal urethra and urinary flow disturbances [1]. Some studies have indicated a relationship between BPH and hypertension [2, 3]. In clinics, medical treatments for BPH are widely used alpha-1 antagonists and 5-alpha-reductase inhibitors. 

However, the side effects, such as postural hypotension, erectile dysfunction, and ejaculatory difficulty, still disturb patients' life qualities [4, 5]. Therefore, understanding the changes of prostatic relaxation in hypertension is helpful.

Loperamide is widely used in clinics for a variety of diarrheal syndromes, including acute and nonspecific (infectious) diarrhea [6, 7]. In recent years, we identified opioid *μ*-receptors expression in rat prostates, and prostatic relaxation was induced by an activation of opioid *μ*-receptors using loperamide [8]. Loperamide has been introduced as the peripheral agonist of opioid *μ*-receptors with poor ability to penetrate the blood-brain barrier [9, 10]. Basically, opioid *μ*-receptor has been divided into 3 subtypes, including *μ*-1, *μ*-2, and *μ*-3 opioid receptors [11]. It has been identified that prostatic relaxation induced by loperamide is mediated through an activation of opioid *μ*-2 receptors [12]. However, change of this prostatic relaxation in hypertension is still unclear.

Otherwise, ATP-sensitive K^+^ (K_ATP_) channels are involved in the relaxation of urethral smooth muscle [13]. Actually, opening of K_ATP_ channel is introduced to lower intracellular Ca^+^ concentration [14, 15]. Moreover, the impairment of K_ATP_ channel may be associated with the dysfunction of lower urinary tract [16]. Actually, K_ATP_ channel is mentioned as the signal in prostatic relaxation induced by loperamide [12]. However, role of K_ATP_ channel in the change of prostatic relaxation in hypertension remains obscure.

In an attempt to clarify the change of prostatic relaxation in hypertension, we used loperamide as agonist to induce relaxation in isolated prostate. Then, we compared the differences of responses to loperamide in prostates isolated from normal and hypertensive rats. Also, signal expressions were investigated to understand the potential mechanism(s) of this change.

## 2. Materials and Methods

### 2.1. Experimental Animals

We obtained 12-week-old male Wistar-Kyoto (WKY) rats and spontaneously hypertensive rats (SHR) from the animal center of National Cheng Kung University Medical College. Rats were maintained in a temperature-controlled room (25 ± 1°C) under a 12 h light-dark cycle (lights on at 06:00). All rats were given water and fed standard chow (Purina Mills, LLC, St Louis, MO, USA) *ad libitum*. All animal-handling procedures were performed according to the *Guide for the Care and Use of Laboratory Animals* of the National Institutes of Health, as well as the guidelines of the Animal Welfare Act.

### 2.2. Preparation of Isolated Prostate Strips

In all prostatic experiments, the isolated prostates from WKY and SHR rats were used. Each rat was killed by decapitation under anesthesia with pentobarbital (50 mg/kg). Following our previous study, the prostate strips were rapidly removed and placed in oxygenated Krebs' buffer (95% O_2_, 5% CO_2_). After the prostate strips had been carefully freed from fat and connective tissue, the strips were then mounted in organ baths filled with 10 mL oxygenated Krebs' buffer (95% O_2_, 5% CO_2_) at 37°C containing (in mmol/L): NaCl 135; KCl 5; CaCl_2_ 2.5; MgSO_4_ 1.3; KH_2_PO_4_ 1.2; NaHCO_3_ 20; d-glucose 10 (pH 7.4).

Each preparation was connected to strain gauges (FT03; Grass Instrument, Quincy, MA, USA). Isometric tension was recorded using chart software (MLS023, Powerlab; ADInstruments, Bella Vista, NSW, Australia). Strips were mounted and allowed to stabilize for 2 h. Each preparation was then gradually stretched to achieve an optimal resting tension of 0.5 g.

### 2.3. Prostatic Relaxation Caused by Loperamide

After the resting tension had stabilized, solution of phenylephrine (Sigma-Aldrich, St Louis, MO, USA) or KCl prepared in distilled water was added into bathing buffer to induce a rapid increase in prostatic tone followed by stable constriction (tonic contraction). The final concentration in the organ bath for phenylephrine was 1 *μ*mol/L and for KCl was 50 mmol/L, respectively. Prostate strips in the treatment group were exposed to loperamide (0.1–10 *μ*mol/L) to observe the decrease in tonic tone (relaxation). Relaxation is expressed as the percentage decrease of maximal tonic contraction. Concentration-relaxation curves were generated in cumulative fashion.

### 2.4. Effects of Blockers on Loperamide-Induced Prostatic Relaxation

Prostate strips were exposed to glibenclamide (Research Biochemical, Wayland, MA, USA) or opioid *μ*-receptor antagonist, cyprodime or naloxonazine (Tocris Cookson, Bristol, UK), for 15 min before the addition of loperamide into organ bath. In addition, the inhibitor of cyclic AMP phosphodiesterase (IBMX) or protein kinase A (H-89) was treated in the same manner. The changes of relaxation caused by loperamide were compared with that in vehicle-(distilled water-) treated controls.

### 2.5. Western Blotting Analysis

The prostate tissues were put in ice-cold homogenized buffer containing 10 mM Tris-HCl (pH 7.4), 20 mM EDTA, 10 mM EGTA, 20 mM *β*-glycerolphosphate, 50 mM NaF, 50 mM sodium pyrophosphate, 1 mM phenylmethylsulfonyl fluoride, and the protease inhibitors 25 *μ*g/mL leupeptin and 25 *μ*g/mL aprotinin. The mixture was centrifuged at 1000 ×g for 10 min at 4°C. The supernatant containing the membrane fraction was centrifuged at 48,000 ×g for 30 min at 4°C. The supernatant was removed, and the pellet was resuspended in Triton X-100 lysis buffer on ice for 30 min, homogenized, and then centrifuged at 14,010 ×g for 20 min at 4°C. Finally, the supernatant was transferred to a new Eppendorf tube and stored at −80°C. The membrane extracts (20–80 *μ*g) were separated by performing SDS-polyacrylamide gel electrophoresis, and the proteins were transferred onto a BioTraceTM polyvinylidene fluoride (PVDF) membrane (Pall Corporation, Pensacola, FL). Following blocking, the blots were developed using antibodies for opioid *μ*-receptors (MOR) (Abcam, Cambridge, UK), sulfonylurea receptor (SUR) (Millipore) or inwardly-rectifying potassium channel (Kir) 6.2 subunits (Kir 6.2) (Santa Cruz Biotechnology, CA). The blots were subsequently hybridized using horseradish peroxidase-conjugated goat anti-goat IgG (Jackson ImmunoResearch Laboratories, Inc., PA), and developed using the Western Lightning Chemiluminescence Reagent PLUS (PerkinElmer Life Sciences Inc., Boston, MA). Densities of the obtained immunoblots at 48 KDa for OMR, 170 KDa for SUR, 40 KDa for Kir 6.2 and 43 KDa for actin were quantified using Gel-Pro analyser software 4.0 (Media Cybernetics, Silver Spring, MD, USA).

### 2.6. Statistical Analysis

All values are presented as the mean ± SEM for a given number of animals or samples. Analysis of variance and Dunnett's post hoc test were used to evaluate the significance between groups. *P* < 0.05 was considered as a significant difference.

## 3. Results

### 3.1. Reduction of Loperamide-Induced Prostatic Relaxation in SHR

**Figure 1:**
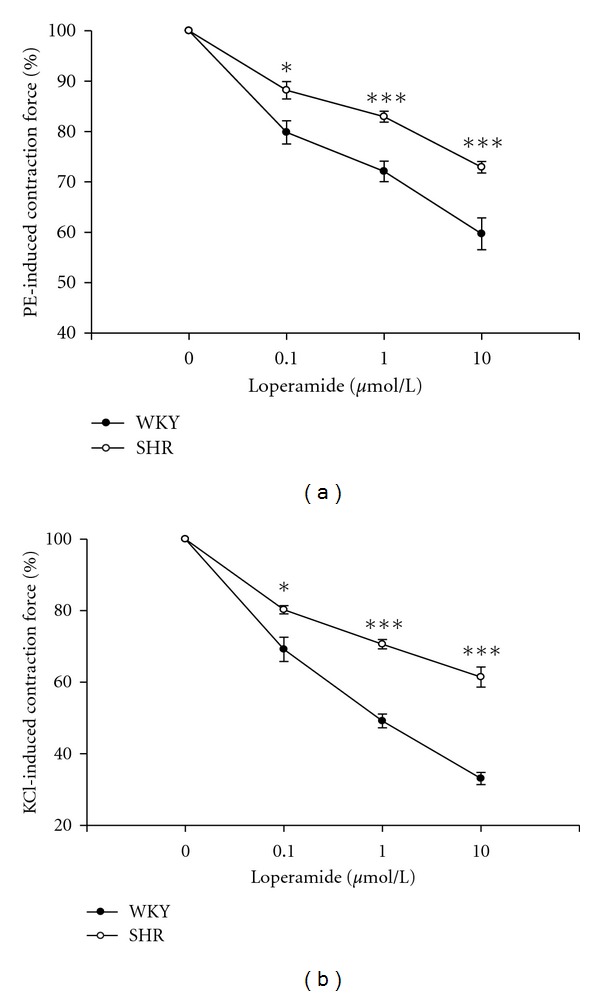
Concentration-dependent relaxation induced by loperamide in isolated prostate strips contracted with 1 *μ*mol/L phenylephrine (a) or 50 mmol/L KCl (b) in WKY and SHR, respectively. Data represent mean ± SEM of eight animals. *P < 0.05, and ***P < 0.001 compared with WKY group.

Prostate strips strongly contracted by the application of phenylephrine (PE) (1 *μ*mol/L) or KCl (50 mmol/L). The prostatic contractions evoked by PE or KCl were not altered in SHR. As shown in Figure 1(a), loperamide relaxed PE-contracted prostate strips from WKY and SHR in a concentration-dependent manner. The effect of loperamide was reversible after washout and repeatable with a second application. Compared to that from WKY, the relaxation of PE-induced prostatic contraction by loperamide in SHR was significantly reduced. Also, the loperamide-induced relaxation in prostate strips precontracted with KCl isolated from SHR was markedly lower than that in WKY (Figure 1(b)). Reduction of loperamide-induced prostatic relaxation in SHR seems more significant in samples contracted with KCl than that with PE. At the maximal concentration tested (10 *μ*mol/L), loperamide significantly attenuated the contraction of SHR prostate strips induced by PE from 59.69 ± 3.54% to 72.91 ± 1.05% of tonic contraction. However, 10 *μ*mol/L loperamide lowered KCl-induced contraction from 33.08 ± 1.89% to 61.45 ± 2.43% of the tonic tone.

### 3.2. Effect of Opioid Receptor Blockade on Loperamide-Induced Prostatic Relaxation

At the maximal concentration (10 *μ*mol/L), loperamide significantly attenuated the tonic contraction of isolated prostate strips induced by PE to 72.91 ± 1.05% in SHR. Also, 10 *μ*mol/L loperamide lowered KCl-induced contraction to 61.45 ± 2.43% of the tonic tone. Then, cyprodime (0.1–1 *μ*mol/L) produced a significant and concentration-dependent attenuation of the relaxant effect of loperamide on tonic contraction of PE-contracted prostate strips from SHR. The prostatic relaxation due to loperamide in KCl-treated prostate strips was also abolished in a similar manner in the presence of cyprodime (Table 1). In addition, naloxonazine failed to abolish the relaxant effect of loperamide on tonic contraction in PE (1 *μ*mol/L)-contracted prostate strips at higher concentration (1 *μ*mol/L). As shown in Table 1, the prostatic relaxation by loperamide in KCl-(50 mmol/L-) contracted prostate strips was also not reversed by naloxonazine even at higher concentration.

### 3.3. Characterization of Signals in Loperamide-Induced Prostatic Relaxation in SHR

In prostate strips of SHR precontracted with phenylephrine (1 *μ*mol/L) or KCl (50 mmol/L), as shown in Table 2, loperamide-induced relaxation was also abolished by pretreatment with glibenclamide (1 *μ*mol/L). Moreover, prostatic relaxation by loperamide was increased by 3-isobutyl-1-methylxanthine (IBMX) at concentration (10 *μ*mol/L) sufficient to inhibit cAMP-phosphodiesterase [17], and decreased by H-89 at concentration (1 *μ*mol/L) enough to abolish the protein kinase A (PKA) [18].

### 3.4. No Change of Opioid *μ*-Receptors in Prostate of SHR

**Figure 2:**
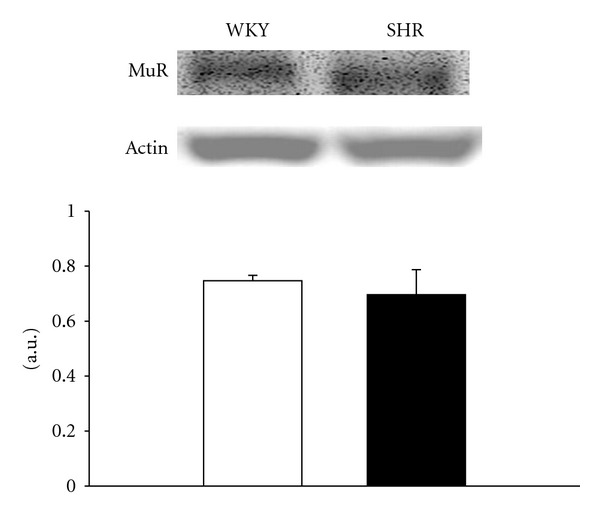
Comparison of the protein level for opioid *μ*-receptor in prostates between WKY and SHR. Data represent mean ± SEM of six animals. There was no difference between WKY and SHR.

The expression of opioid *μ*-receptors in prostates from SHR was similar to that from WKY (Figure 2). Quantification of the protein levels was shown in Figure 2 and no difference can be obtained in samples between WKY and SHR.

### 3.5. Reduction of Diazoxide-Induced Prostatic Relaxation in SHR

**Figure 3:**
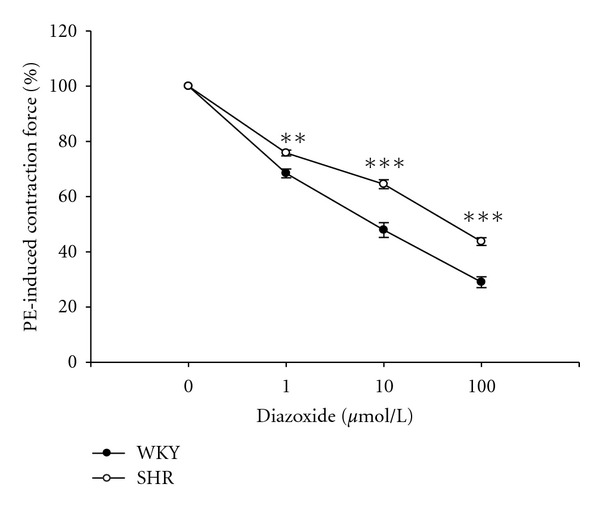
Concentration-dependent relaxation of diazoxide in isolated SHR prostates contracted with 1 *μ*mol/L phenylephrine. Data represent mean ± SEM of eight animals. **P < 0.01 and ***P < 0.001 compared with WKY group.

As shown in Figure 3, diazoxide relaxed PE-contracted prostate strips from WKY and SHR in a concentration-dependent manner. Compared to that from WKY, the relaxation of PE-induced prostatic contraction by diazoxide in SHR was significantly reduced.

### 3.6. Changes of Potassium Channels (SUR and Kir 6.2) in Prostate of SHR

**Figure 4:**
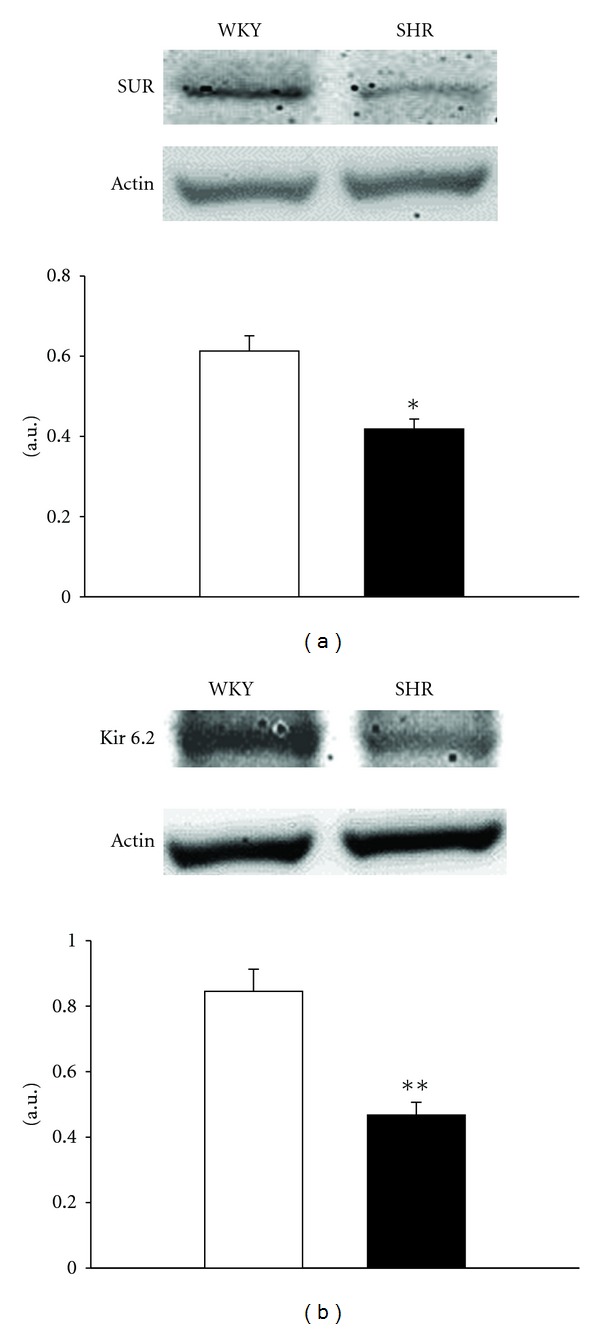
The difference in the protein levels of sulphonylurea receptors (SUR) and inwardly rectifying K^+^ channel subunit 6.2 (Kir 6.2) obtained from prostates between WKY and SHR. Data represent mean ± SEM of six animals. *P < 0.05 and **P < 0.01 compared with WKY group.

The expressions of SUR and Kir 6.2 in prostates from SHR were significantly decreased as compared with that from WKY (Figure 4). Quantification of the protein levels was also shown in Figure 4.

## 4. Discussion

In the present study, we found that prostatic relaxation caused by loperamide is markedly reduced in SHR as compared to that in WKY. The dose-dependent relaxation induced by loperamide was observed in prostate strips contracted with PE or KCl. Also, the decrease of loperamide-induced prostatic relaxation seems more significant in KCl-contracted samples than that in PE-contracted samples. Thus, it is of special interesting to understand the potential mechanism(s) of this difference.

The action of loperamide is mostly related to the activation of opioid receptors in peripheral tissue because loperamide is hard to enter into central nervous system [10, 19]. In the present study, the action of loperamide is effectively abolished by cyprodime at a concentration sufficient to block opioid *μ*-receptors, suggesting an activation of opioid *μ*-receptors by loperamide in bladder relaxation of SHR. However, the action of loperamide was not reversed by naloxonazine even at a concentration sufficient to block opioid *μ*-1 receptors. Mediation of opioid *μ*-1 receptors seems unlikely in the prostatic relaxation of loperamide caused in SHR. In recent, opioid *μ*-receptor has been divided into 3 subtypes, including *μ*-1, *μ*-2, and *μ*-3 opioid receptors [20–22]. The activation of opioid *μ*-1 receptors seems related to smooth muscle contraction via PLC-PKC pathway [23, 24]. Also, opioid *μ*-3 receptors are mostly presented in endothelial cells associated with the production of nitric oxide to induce vasodilatation [25]. Therefore, mediation of opioid *μ*-1 or *μ*-3 receptors in prostatic relaxation seems unlikely. Moreover, activation of opioid *μ*-2 receptors participated in the relaxation of guinea pig ileum and inhibition of gastrointestinal transit [26, 27]. Taken together, an activation of opioid *μ*-2 receptors is more reliable to participate in the action of loperamide for relaxation of prostate strips isolated from SHR. This result is consistent with our previous report in normal rats [12]. The concentration of loperamide tested in the present study is sensitive to the receptor sites especially in the isolated preparation. The concentration for a clinical situation is hard to calculate from the dose used in animal only and it should be really monitored in human.

However, the decrease of prostatic relaxation induced by loperamide seems not to be related to the change of opioid *μ*-receptors in SHR because there was no difference in protein level of opioid *μ*-receptors between SHR and WKY identified by western blotting analysis (Figure 2).

Otherwise, prostatic relaxation by loperamide in SHR was attenuated by blockade of ATP-sensitive K^+^ (K_ATP_) channels, indicating the involvement of K_ATP_ channels in prostatic relaxation by loperamide. Potassium channels playing an important role in the regulation of prostatic contractility has been indicated in guineapig [28]; the activation of K_ATP_ channels causes hyperpolarization of cell membrane and consequently relaxes smooth muscle. It has been established that an activation of adenylyl cyclase can increase the intracellular cyclic AMP (cAMP) to activate cAMP-dependent protein kinase (PKA) for opening of K_ATP_ channels [18]. As shown in Table 2, we characterized that loperamide-induced prostatic relaxation was blocked by glibenclamide. The prostatic relaxation of loperamide was abolished by H-89 at the concentration sufficient to block PKA [18] and enhanced by IBMX at concentration enough to inhibit cAMP-posphodiesterase [17]. These data suggest that the possible mechanism for loperamide-induced prostatic relaxation in SHR is mediated through cAMP-PKA pathway to open K_ATP_ channels, which is consistent with the previous phenomenon for loperamide-induced prostatic relaxation in normal rats [12]. Thus, we focused on the role of K_ATP_ channels in the change of prostatic relaxation by loperamide in SHR.

We used diazoxide the well-known agent as potassium channel opener [29] to investigate the changes of action in SHR. Similar to the previous report [30], diazoxide induced a dose-dependent relaxation in prostate contracted with PE. Prostatic relaxation caused by diazoxide was also reduced in samples from SHR as compared to that from WKY (Figure 3). Role of potassium channels in the change of prostatic relaxation by loperamide in SHR can be considered. A reduction of potassium channel has been observed in human prostate cancer [31].

Moreover, the ATP-sensitive K^+^ (K_ATP_) channels are composed of four inwardly rectifying K^+^ channel (Kir) subunits and four regulatory sulphonylurea receptors (SUR) [32]. In the present study, we found that expressions of Kir and SUR in prostate tissues are both lowered in SHR (Figure 4). A decrease of K_ATP_ channels in prostate of SHR can thus be identified; this is consistent with the reduction of prostatic relaxation caused by diazoxide in SHR. Also, the relaxation of loperamide in prostate strips of SHR was abolished by the pretreatment with glibenclamide at concentration sufficient to block K_ATP_ channels. Therefore, decrease of K_ATP_ channels is important in the reduction of prostatic relaxation induced by loperamide in SHR; the obtained results provide novel insight into the potential mechanisms that were not mentioned before. In the present study, prostate relaxation induced by loperamide was reduced in relation to the changes of potassium channels expression under hypertensive condition. Many factors can be involved in this change, such as the continuous high blood pressure or endogenous substance(s) induced by hypertension and/or others. However, the real mechanism(s) remain obscure and it requires more experiments to clarify in advance. Moreover, the role of K_ATP_ channels is mainly focused for prostatic relaxation in this study. Actually, other targets are possibly involved in the mechanism for potential drug development, such as phosphodiesterase V [33] and cyclic GMP-dependent protein kinase-1 [34]. However, the improvement of hypertension would be helpful in the treatment of BPH. Thus, regular control of blood pressure is important in the prevention of prostatic damage.

## 5. Conclusions

In conclusion, we suggest that the dysfunction of K_ATP_ channels explains poor prostatic relaxation-induced by loperamide under hypertensive condition. Therefore, improvement of prostatic K_ATP_ channels will be a new target in the development of agents for handling BPH in hypertensive patients.

## Figures and Tables

**Table 1:** The inhibitory effect of cyprodime or naloxonazine on the relaxation of loperamide (10 *μ*mol/L) in isolated SHR prostates contracted with 1 *μ*mol/L phenylephrine (PE) or 50 mmol/L KCl. Data represent mean ± SEM of eight animals.

	PE (%)	KCl (%)
Loperamide (10 *μ*mol/L)		
+ Vehicle	72.91 ± 1.05	61.45 ± 2.43
+ Cyprodime		
0.1 *μ*mol/L	81.23 ± 0.71***	78.92 ± 0.76**
1.0 *μ*mol/L	92.60 ± 1.30***	86.68 ± 1.98***
+ Naloxonazine		
0.1 *μ*mol/L	70.62 ± 1.02	60.77 ± 1.00
1.0 *μ*mol/L	70.19 ± 2.17	63.79 ± 1.92

**P < 0.01 and ***P < 0.001 compared with vehicle-treated control.

**Table 2:** The effects of inhibitors for signals on the relaxation induced by loperamide (10 *μ*mol/L) in SHR isolated prostates contracted with 1 *μ*mol/L phenylephrine (PE) or 50 mmol/L KCl. Data represent mean ± SEM of eight animals.

	PE (%)	KCl (%)
Loperamide (10 *μ*mol/L)		
+ Vehicle	73.53 ± 1.06	63.17 ± 2.55
+ IBMX (10 *μ*mol/L)	62.16 ± 1.62**	51.20 ± 1.18*
+ H-89 (1 *μ*mol/L)	86.42 ± 1.59***	81.66 ± 1.62**
+ Glibenclamide (1 *μ*mol/L)	93.21 ± 0.62***	85.02 ± 0.68***

*P < 0.05, **P < 0.01 and ***P < 0.001 compared with vehicle-treated control, respectively.
